# Genetic polymorphisms involved in dopaminergic neurotransmission and risk for Parkinson's disease in a Japanese population

**DOI:** 10.1186/1471-2377-11-89

**Published:** 2011-07-25

**Authors:** Chikako Kiyohara, Yoshihiro Miyake, Midori Koyanagi, Takahiro Fujimoto, Senji Shirasawa, Keiko Tanaka, Wakaba Fukushima, Satoshi Sasaki, Yoshio Tsuboi, Tatsuo Yamada, Tomoko Oeda, Hiroyuki Shimada, Nobutoshi Kawamura, Nobutaka Sakae, Hidenao Fukuyama, Yoshio Hirota, Masaki Nagai

**Affiliations:** 1 Department of Preventive Medicine, Graduate School of Medical Sciences, Kyushu University, Fukuoka, Japan; 2 Department of Preventive Medicine and Public Health, Faculty of Medicine, Fukuoka University, Fukuoka, Japan; 3 Department of Cell Biology, Faculty of Medicine, Fukuoka University, Fukuoka, Japan; 4 Department of Public Health, Osaka City University Graduate School of Medicine, Osaka, Japan; 5 Department of Social and Preventive Epidemiology, School of Public Health, The University of Tokyo, Tokyo, Japan; 6 Department of Neurology, Faculty of Medicine, Fukuoka University, Fukuoka, Japan; 7 Clinical Research Institute and Department of Neurology, Utano National Hospital, Kyoto, Japan; 8 Department of Geriatrics and Neurology, Osaka City University Graduate School of Medicine, Osaka, Japan; 9 Department of Neurology, Neurological Institute, Graduate School of Medical Sciences, Kyushu University, Fukuoka, Japan; 10 Human Brain Research Center, Kyoto University Graduate School of Medicine, Kyoto, Japan; 11 Department of Public Health, Saitama Medical University Faculty of Medicine, Saitama, Japan; 12 Other members of the Study Group are listed in the Appendix

## Abstract

**Background:**

Parkinson's disease (PD) is characterized by alterations in dopaminergic neurotransmission. Genetic polymorphisms involved in dopaminergic neurotransmission may influence susceptibility to PD.

**Methods:**

We investigated the relationship of catechol-O-methyltransferase (COMT), monoamine oxidase B (MAOB), dopamine receptor (DR) D2 and DRD4 polymorphisms and PD risk with special attention to the interaction with cigarette smoking among 238 patients with PD and 369 controls in a Japanese population.

**Results:**

Subjects with the AA genotype of *MAOB *rs1799836 showed a significantly increased risk of PD (odds ratio (OR) = 1.70, 95% confidence interval (CI) = 1.12 - 2.58) compared with the AG and GG genotypes combined. The AA genotype of *COMT *rs4680 was marginally associated with an increased risk of PD (OR = 1.86, 95% CI = 0.98 - 3.50) compared with the GG genotype. The *DRD2 *rs1800497 and *DRD4 *rs1800955 polymorphisms showed no association with PD. A *COMT *-smoking interaction was suggested, with the combined GA and AA genotypes of rs4680 and non-smoking conferring significantly higher risk (OR = 3.97, 95% CI = 2.13 - 7.41) than the AA genotype and a history of smoking (P for interaction = 0.061). No interactions of smoking with other polymorphisms were observed.

**Conclusions:**

The *COMT *rs4680 and *MAOB *rs1799836 polymorphisms may increase susceptibility to PD risk among Japanese. Future studies involving larger control and case populations and better pesticide exposure histories will undoubtedly lead to a more thorough understanding of the role of the polymorphisms involved in the dopamine pathway in PD.

## Background

Dopamine is one of the major modulatory neurotransmitters in the central nervous system (CNS) [1]. As dysfunction of dopaminergic neurotransmission in the CNS has been implicated in development of PD [2], it has been suggested that genetic polymorphisms involved in the biosynthesis and degradation of dopamine and related compounds influence susceptibility to PD. Catechol-O-methyltransferase (COMT) is an enzyme, which by methylation inactivates neurotransmitters and toxic catechols such as the immediate precursor of dopamine. Monoamine oxidase B (MAOB) is one of the primary enzymes regulating metabolism of neurotransmitters such as dopamine. There are five known dopamine receptors (DRD1-5) grouped into D-1 like (DRD1 and DRD5) and D-2 like (DRD2, DRD3 and DRD4) receptors based on their pharmacological profiles and sequence homology. Of these, DRD2 and DRD4 govern the signaling effect and modulate the motor behavior and activity of nigrostriatal neurons [3]. Genetic variation in these proteins, which are responsible for dopaminergic neurotransmission, may influence susceptibility to PD.

Decreased COMT activity may result in increased metabolism of dopamine to neuromelanin that can enhance the formation of cyototoxic radicals contributing to neuronal degeneration [4]. As the A allele of *COMT *rs4680 is associated with low COMT activity of soluble COMT [5], the A allele of *COMT *rs4680 may be linked to an increased risk of PD. It has been suggested that MAOB inhibition may prevent degeneration of the dopaminergic system in PD [6]. It is well-documented that cigarette smoking is associated with reduced MAOB activity and confers beneficial effects against PD [7]. Therefore, low MAOB activity may play a preventive role in PD development. Although the *MAOB *rs1799836 polymorphism is a synonymous substitution, this single nucleotide polymorphism (SNP) is associated with varying enzyme activity. In fact, synonymous SNPs can cause inactivation of the native splicing donor site, which results in a premature stop codon or exon skipping, yielding a shorter mRNA [8]. The shorter mRNA results in a truncated protein that is likely rapidly degraded or functionally inactive [9]. As the G allele of *MAOB *rs1799836 polymorphism is associated with lower activity of brain MAOB activity [10], the G allele may be involved in PD susceptibility (protective). The *DRD2 *rs1800497 T allele (formerly *DRD2 *TaqI A1) showed reduced DRD2 density in the postmortem brain [11], decreased receptor binding in positron emission tomography *in vivo *[12] and reduction of dopaminergic activity in the CNS [13]. However, the impact of *DRD2 *rs1800497 on D2 receptor density has recently been questioned [14]. The functional significance of *DRD2 *rs1800497 is not clear at this time, and there may be linkage disequilibrium between the other polymorphisms. The *DRD4 *rs1800955 SNP is thought to influence promoter activity with the T allele exhibiting a 40% reduction in promoter activity relative to the C allele *in vitro *[15]. As the T allele of *DRD4 *rs1800955 is considered to be involved in defects in dopaminergic neurotransmission, the T allele may play a deleterious role in PD development.

Studying gene-environment interactions in relation to PD risk may be valuable because positive findings would clearly implicate disease-causing exposures, clarify PD etiology, and elucidate environmental modifications for disease prevention. This study aimed to determine the impact of polymorphisms involved in dopaminergic neurotransmission on PD risk alone or in combination with smoking in a Japanese population.

## Methods

### Study subjects

PD patients were recruited at three university hospitals and one national hospital in Fukuoka Prefecture, a metropolitan area of Kyushu Island in southern Japan, and in three university hospitals, three national hospitals and one municipal hospital in Osaka, Kyoto, and Wakayama Prefectures. Eligible (prevalent) cases were patients who were within 6 years of the onset of PD and who presented at one of the 11 collaborating hospitals between April 1, 2006 and March 31, 2008. The mean duration (± SD) of PD was 38.8 (16.7) months. The mean age of onset (± SD) was 65.76 (± 8.82) years. There were no patients with juvenile PD. During the same period, hospital controls, without a previous diagnosis of a neurodegenerative or malignant disease, were recruited from departments other than the department of neurology because hospital controls are more motivated and are more easily accessible for obtaining DNA samples. Controls were not, individually or in larger groups, matched to cases. Details of the study subjects have been documented elsewhere [13].

Six hundred and eleven subjects (240 PD patients and 371 controls) agreed to donate buccal samples. Data on smoking and pesticide use were insufficient for two cases and one control. In total, 238 cases and 369 controls were enrolled in this study. The ethics committees of the eleven collaborating universities/hospitals approved the research protocol, and all subjects signed informed consent.

### Genetic analysis

Genomic DNA was extracted from buccal samples. Genetic determinations were blinded to PD status. TaqMan SNP Genotyping Assays (ABI) were used for the following (gene, SNP, assay ID): *COMT*, rs4680, C_25746809_50; *MAOB*, rs1799836, C_8878790_10; *DRD2*, rs1800497, C_7486676_10; *DRD4*, rs1800955, C_7470700_30.

### Statistical analysis

To test for associations between SNPs and PD, we defined the ancestral allele using the National Center for Biotechnology Information SNP database as the major allele. We assessed Hardy-Weinberg equilibrium (HWE) via a goodness-of-fit χ^2 ^test (Pearson) to compare the observed and expected genotype frequencies among controls. Based on the results from functional studies (SNPs other than *DRD2 *rs1800497) and our results of associations between SNPs and PD, we designated the genotype presumed to increase the risk of PD as the "at-risk" genotype. The trend of association was assessed by a logistic regression model assigning ordinal scores to the levels of the independent variable. As *MAOB *is located on the X chromosome (Xp11.23), the genotypes were assessed separately in men and women. Although men are in a hemizygous state, the genotypes of *MAOB *rs1799836 for men were coded as homozygous. All "at-risk" alleles were classified into six categories (0-2 and 3, 4, 5, 6, and 7+). Alternatively, all "at-risk" alleles were classified into four categories (0-3 and 4, 5, 6 +). Unconditional logistic regression was used to compute the odds ratios (ORs) and their 95% confidence intervals (CIs), with adjustments for potential confounders. The potential confounders included age (continuous variable), sex (male/female), region of residence (Fukuoka/Kinki), smoking status (ever/never), alcohol consumption [long-term consumption of alcoholic beverages (continuing consuming for ≥49 years, which is a cutoff point at the 90th percentile of controls)/short-term consumption of alcoholic beverages (continuing consuming for < 49 years)] and pesticide, herbicide or fungicide exposure (ever/never). Modeling different mechanisms of action of a particular allele was conducted by grouping individuals with one or two particular genotypes regarding the chosen model (dominant model: scored as 1 for heterozygous and homozygous of the possible risk allele for PD and 0 otherwise; recessive model: scored as 1 for homozygous of the possible risk allele for PD and 0 otherwise). The interaction between SNPs and cigarette smoking on the risk of PD was statistically evaluated based on the likelihood test, comparing the models with and without a term for interaction (multiplicative scale).

All statistical analyses were implemented in STATA Version 10.1. All P values were two-sided, with those less than 0.05 considered statistically significant.

## Results

The distributions of selected characteristics among subjects are summarized in Table 1. Two hundred and thirty-eight patients with PD and 369 controls were enrolled in the study. The sex ratio, the prevalence of first degree family history of PD and the region of residence did not differ significantly between cases and controls. Compared with control subjects, cases were more likely to be older (P = 0.007) and report long-term alcohol consumption (P = 0.041). PD patients were less likely to report a history of smoking compared to the control subjects (P < 0.0001). Unexpectedly, the PD patients tended to have less frequent home or occupational pesticide exposure.

**Table 1:** Selected characteristics of Parkinson's disease cases and controls

Characteristics	Cases (n = 238)	Controls (n = 369)	*P*
Age, year (95% CI)	68.5 (67.4 - 69.6)	66.6 (65.7 - 67.4)	0.007
Sex, n (%)			
Male	91 (38.2)	140 (38.0)	
Female	147 (61.8)	228 (62.0)	0.96
First degree family			
history of PD, n (%)	11 (4.62)	12 (3.25)	0.39
Smoking status, n (%)			
Current smoker	7 (2.94)	50 (13.6)	
Former smoker	57 (24.0)	97 (26.3)	
Non-smoker	174 (73.1)	222 (60.2)	< 0.0001
Consumption of alcoholic			
beverages, n (%)*			
Short-term	195 (81.9)	320 (87.9)	
Long-term	43 (18.1)	44 (12.1)	0.041
Home pesticide use, n (%)			
Yes	117 (49.2)	202 (54.7)	
No	121 (50.8)	167 (45.3)	0.18
Occupational pesticide use, n (%)			
Yes	20 (8.4)	33 (8.9)	
No	218 (91.6)	336 (91.1)	0.82
Either home or occupational			
pesticide use, n (%)			
Yes	122 (51.3)	210 (56.9)	
No	116 (48.7)	159 (43.1)	0.17
Region of residence, n (%)			
Fukuoka	89 (37.4)	154 (41.7)	
Kinki	149 (62.6)	215 (58.3)	0.29

95% CI, 95% confidence interval

Five cases were missing.

The distributions of polymorphisms involved in dopaminergic neurotransmission among cases and controls are shown in Table 2. Four SNPs did not deviate from HWE in controls (P_HWE _= 0.077 for *COMT *rs4680, P_HWE _= 0.443 for *MAOB *rs1799836 among women, P_HWE _= 0.111 for *DRD2 *rs1800497, P_HWE _= 0.083 for *DRD4 *rs1800955). As *MAOB *is located on the X chromosome, rs1799836 among men (no heterozygotes) and women combined deviated from HWE. There were nonsignificant differences in genotypic frequencies between case and control subjects for all of the polymorphisms (P = 0.106 - 0.460). The AA genotype of *COMT *rs4680 was marginally associated with an increased risk of PD compared with the GG genotype (OR = 1.86, 95% CI = 0.98 - 3.50). There was a significant trend in increasing risk with the number of the A alleles of *COMT *rs4680 (P_trend _= 0.044). A dominant effect of the A allele on PD risk was suggested. Subjects with the AA genotype had a significantly increased risk of PD compared with those with at least one G allele (adjusted OR = 1.70, 95% CI = 1.12 - 2.58). There was a significant trend in decreasing risk with the number of the G alleles of *MAOB *rs1799836 (P_trend _= 0.016). A recessive effect of the A allele of *MAOB *rs1799836 on PD risk was suggested. The C allele of *DRD2 *rs1800497 was associated with an increased risk of PD and appeared to act in a recessive mode in this study. Similarly, the T allele of *DRD4 *rs1800955 was regarded as the putative risk allele and appeared to behave in a dominant fashion.

**Table 2:** Associations of polymorphisms involved in dopaminergic neurotransmission and Parkinson's disease

Polymorphism	Cases (%) (n = 238)	Controls (%) (n = 369)	P	Adjusted* OR (95% CI)
*COMT *rs4680				
GG (ancestral)	98 (41.2)	179 (48.5)		1.0
GA	116 (48.7)	166 (45.0)		1.26 (0.88 - 1.79)
AA	24 (10.1)	24 (6.5)	0.106	1.86 (0.98 - 3.50)
		P_HWE _= 0.077		P_trend = _0.044
GA + AA vs. GG	140 (58.8)	190 (51.4)		1.33 (0.95 - 1.87)
				
*MAOB *rs1799836				
AA (A) (ancestral)	192 (80.7)	273 (74.0)		1.0
AG	34 (14.3)	68 (18.4)		0.61 (0.37 - 0.99)
GG (G)	12 (5.0)	28 (7.6)		0.55 (0.26 - 1.16)
		P_HWE _< 0.0001	0.154	P_trend = _0.016
AA (A) vs. AG + GG (G)				1.70 (1.12 - 2.58)
Female				
AA (ancestral)	110 (74.8)	156 (68.1)		1.0
AG	34 (23.1)	68 (29.7)		0.60 (0.36 - 0.99)
GG	3 (2.13)	5 (2.18)		0.90 (0.21 - 3.97)
		P_HWE _= 0.443	0.368	P_trend = _0.084
Male				
A	82 (90.1)	117 (83.7)		1.0
G	9 (9.89)	23 (16.3)	0.166	0.45 (0.19 - 1.09)
				
*DRD2 *rs1800497				
TT (ancestral)	29 (12.2)	52 (14.1)		1.0
TC	117 (49.2)	192 (52.0)		1.04 (0.61 - 1.77)
CC	92 (38.7)	125 (33.9)	0.460	1.32 (0.77 - 2.28)
		P_HWE _= 0.111		P_trend = _0.204
CC vs. TC + TT				1.28 (0.90 - 1.82)
				
*DRD4 *rs1800955**				
TT (ancestral)	81 (34.0)	136 (37.0)		1.0
TC	122 (51.3)	162 (44.0)		1.23 (0.85 - 1.79)
CC	35 (14.7)	70 (19.0)	0.173	0.88 (0.54 - 1.47)
		P_HWE _= 0.083		P_trend = _0.928
TT + TC vs. CC				1.27 (0.80-2.00)

*Adjusted for age, sex, first degree family history of PD, region, smoking status, drinking status and pesticide exposure.

**One control was missing.

We assessed interactions between polymorphisms involved in dopaminergic neurotransmission and smoking (Table 3). To achieve adequate statistical power, current and former smokers were combined (ever smokers). The OR of a history of smoking was 0.40 (95% CI = 0.25 - 0.64) after adjustment for age, sex, first degree family history of PD, region, alcohol consumption and pesticide use (data not shown). As shown in Table 3, non-smokers with at least one A allele of *COMT *rs4680 (adjusted OR = 3.97, 95% CI = 2.13 - 7.41) had a higher risk of PD than those with the GG genotype (adjusted OR = 3.70, 95% CI = 1.95 - 7.02), relative to ever smokers with the GG (non-risk) genotype (reference). Ever smokers with the GA and AA genotypes combined had a significantly increased risk of PD (adjusted OR = 2.19, 95% CI = 1.17 - 4.10). Evidence of interaction between the *COMT *rs4680 genotypes and smoking was suggested (P = 0.061). Similarly, non-smokers with the "at-risk" AA genotype of *MAOB *rs1799836 (adjusted OR = 5.74, 95% CI = 2.16 - 15.2) had a higher risk of PD than those with the AG and GG genotypes combined (adjusted OR = 3.68, 95% CI = 1.30 - 10.4), relative to ever smokers with the AG and GG genotypes combined. Smokers with the AA genotype presented a nonsignificantly increased risk of PD (adjusted OR = 2.39, 95% CI = 0.91 - 6.27). Interaction between the *MAOB *rs1799836 genotypes and smoking was far from significant. As for the *DRD2 *rs1800497 and *DRD4 *rs1800955 SNPs, the significantly high ORs were attributed largely to the effect of non-smoking. The interaction measure between smoking and either *DRD2 *rs1800497 or *DRD4 *rs1800955 did not reach statistical significance.

**Table 3:** Interaction between smoking and polymorphisms involved in dopaminergic neurotransmission in Parkinson's disease

Polymorphism	Genotype	Non-smokers		Ever smokers		P*_inteaction_*
			
		Cases/controls	Adjusted OR* (95% CI)	Cases/controls	Adjusted OR* (95% CI)	
*COMT*	No risk† (GG)	77/103	3.70 (1.95 - 7.02)	21/76	1.0 (reference)	0.061
rs4680	At-risk† (GA + AA)	97/119	3.97 (2.13 - 7.41)	43/71	2.19 (1.17 - 4.10)	
*MAOB*	No risk‡ [GG (G) + AG]	40/70	3.68 (1.30 - 10.4)	6/26	1.0 (reference)	0.434
rs1799836	At- risk‡ [AA (A)]	134/152	5.74 (2.16 - 15.2)	58/121	2.39 (0.91 - 6.27)	
*DRD2*	No risk‡ (TC + TT)	108/153	2.32 (1.34 - 3.99)	38/91	1.0 (reference)	
rs1800497	At- risk‡ (CC)	66/69	3.16 (1.75 - 5.70)	26/56	1.12 (0.61 - 2.08)	0.608
*DRD4*	No risk† (CC)	25/37	2.98 (1.18 - 7.56)	10/33	1.0 (reference)	
rs1800955**	At- risk† (TT + TC)	149/184	3.50 (1.57 - 7.80)	54/114	1.48 (0.67 - 3.28)	0.637

*Adjusted for age, sex, first degree family history of PD, region, drinking status and pesticide exposure.

**One control was missing.

† Based on the dominant model.

‡ Based on the recessive model.

There were no polymorphism-polymorphism interactions in any possible combination (data not shown).

We examined the cumulative effect of putative "at-risk" alleles of four SNPs involved in dopaminergic neurotransmission on PD risk (Table 4). Increasing numbers of putative "at-risk" alleles increased PD risk in a dose dependent manner (P_trend _= 0.007). The risk was more than doubled in subjects with seven or eight putative "at-risk" alleles (adjusted OR = 2.66, 95% CI = 1.03 - 6.88), compared to those with one or two putative "at-risk" alleles. Alternatively, for carriers with more than five putative "at- risk" alleles, PD risk was increased ~2-fold (OR = 1.80, 95% CI = 1.07 - 3.05), compared with carriers with less than or equal to three putative "at- risk" alleles.

**Table 4:** Relationship of total number of "at-risk" genotypes of polymorphisms involved in dopaminergic neurotransmission to Parkinson's disease

Number of "at-risk"* alleles	Subjects, n (%)		Adjusted† OR (95% CI)		
				
	Cases (n = 238)	Controls** (n = 368)			
0	0 (0.0)	0 (0.0)	-	-	-
1	2 (0.84)	6 (1.63)	1.0 (reference)	1.0	
2	8 (3.36)	16 (4.35)	1.41 (0.22 - 9.06)	(reference)	1.0 (reference)
3	25 (10.5)	46 (12.5)	1.59 (0.29 - 8.74)	1.23 (0.49 - 3.09)	
4	51 (21.4)	100 (27.2)	1.65 (0.31 - 8.78)	1.28 (0.54 - 2.99)	1.10 (0.64 - 1.90)
5	73 (30.7)	107 (29.1)	2.13 (0.41 - 11.2)	1.64 (0.71 - 3.79)	1.42 (0.85 - 2.39)
6	51 (21.4)	65 (17.7)	2.39 (0.45 - 12.7)	1.84 (0.78 - 4.36)	
7	25 (10.5)	26 (7.07)	3.47 (0.62 - 19.4)	2.66	1.80 (1.07 - 3.05)
8	3 (1.26)	2 (0.54)	3.25 (0.28 - 37.8)	(1.03 - 6.88)	
			*P_trend_*= 0.007	*P_trend_*= 0.007	*P_trend_*= 0.012

* Based on our results, we designated the allele that is presumed to increase the risk of PD as the "at-risk" allele.

**One control was missing.

†Adjusted for age, sex, first degree family history of PD, region, smoking status, drinking status and pesticide exposure.

## Discussion

The polymorphisms involved in dopaminergic neurotransmission such as *COMT *rs4680, *MAOB *rs1799836, *DRD2 *rs1800497 and *DRD4 *rs1800955 were determined in a total of 607 Japanese subjects (238 PD cases and 369 controls). As compared with the GG genotype of *COMT *rs4680, the AA genotype was marginally associated with an increased risk of PD. The AA genotype of *COMT *rs4680 has been reported to be a genetic risk factor for PD in Japanese populations [16,17] but the studies among ethnic populations other than Japanese failed to confirm any significant association [5,18-25]. This ethnic difference might be partly due to the difference in the allelic frequency of *COMT *rs4680. According to the HapMap SNP database [26], the A allele frequency is more common among Caucasians (51.7%) and less common among Japanese (23.3%), Han Chinese (25.6%) and Yorubas (a West African ethnic group, 29.2%). The frequency of the A allele in this study (29.0%) was somewhat higher than that of the HapMap SNP database but similar to that of other Japanese populations (28.8% and 31.1%) [16,17]. Generally, the low frequency of the "at risk" allele reduces the statistical power. As the prevalence of the A allele was lower in Japanese than in Caucasians, this is not the case. Given the lower frequency of the A allele in Japanese subjects, if this allele is associated with an increased risk of PD, then the prevalence of PD would be lower among Japanese than Caucasians. In fact, the prevalence of PD is generally lower in Asian and African-American populations than in Caucasian populations [27,28]. The reason why Japanese studies found a significant association between *COMT *rs4680 and PD risk is not clear. The ethnic difference may reflect different gene-environment interactions, gene-gene interactions, or different linkages to the polymorphisms determining PD risk.

As compared with individuals with at least one G allele of *MAOB *rs1799836, those with the AA genotype had a significantly increased risk of PD. A number of studies have examined *MAOB *rs1799836 and PD risk with conflicting results. Some studies reported that the G allele of *MAOB *rs1799836 was significantly associated with an increased risk of PD [22,29,30]. Similarly, the presence of the G allele was associated with a modest increased risk of PD [22,25,31]. On the contrary, a significant association between the A allele of *MAOB *rs179986 and an increased PD risk was observed [32]. Other studies found no association between this polymorphism and PD risk [23,24,33-36]. A meta-analysis based on six studies published before November 1999 reported that there was no significant association of *MAOB *rs1799836 with PD [37]. Each population may have its own set of environmental and genetic factors that contribute to PD risk. The lack of replication can in part be accounted for as the role of *MAOB *rs179986 on PD risk differs with environmental factors such as smoking.

The *DRD*2 rs1800497 and *DRD4 *rs1800955 SNPs were not associated with PD risk in this study. No significant association of *DRD2 *rs1800497 and PD risk has been reported in different populations [30,36,38-40]. However, two studies of Caucasians found that the T allele of *DRD2 *rs1800497 was associated with a significantly increased risk of PD [41,42]. As the conflicting results may be attributed to linkage disequilibrium (LD) between the other polymorphisms, there is a possibility that other polymorphisms such as -141 Ins/Del (rs1799732), Ser311Cys (rs1801028), Taq IB (rs1079597) and C957T (rs6277), which may be in LD with rs1800497, may play a causative role in PD development. The differences in LD would be observed among different populations [43] and different historical stages of the same population [44]. Therefore, it is more likely that the ethnic differences of the association between *DRD2 *rs1800497and PD exist. As reproducibility of the results is important in genetic association studies, additional studies with a large sample size are needed to clarify the pivotal role of *DRD2 *rs1800497 in PD development. Furthermore, the association of the T allele of *DRD2 *rs1800497 with receptor availability was not always replicated. Future mechanistic studies are needed to verify the functional significance of different *DRD2 *rs1800497 alleles. To the best of our knowledge, no studies on the association between *DRD4 *rs1800955 and PD have been previously reported. PD risk associated with the 48 bp tandem repeat polymorphism of *DRD4 *at the third exon, which may also be functional, has been evaluated, and one [45] of four studies [45-48] found a significant association. This tandem repeat polymorphism is probably not the main determinant in developing PD. Again, testing replication in different populations is an important step. Additional studies are warranted to corroborate the null association among Japanese samples suggested in the present study.

It is widely accepted that PD development requires environmental factors acting on a genetically predisposed individual. A gene-environment interaction was suggested, with the GA and AA combined genotype of *COMT *rs4680 and non-smoking conferring significantly higher risk (OR = 3.97, 95% CI = 2.13 - 7.41), compared with the GG genotype and a history of smoking (P for interaction = 0.061). In other words, the impact of the combined genotype of GA and AA on PD risk was marginally different between ever smokers (2.19) and non-smokers (3.97/3.70 = 1.07). In contrast to our results, two studies have reported no interaction between cigarette smoking and *COMT *rs4680 in relation to PD risk [23,25]. Our results suggest that interaction between *COMT *rs4680 and smoking is likely to vary in different races. Additional epidemiological studies are warranted to determine the smoking-*COMT *polymorphism interaction. There were no interactions between *MAOB *rs1799836, *DRD2 *rs1800497 or *DRD4 *rs1800955 polymorphisms and smoking. Conflicting results regarding the modifying effect for *MAOB *rs1799836 on PD risk have been reported. *MAOB *rs1799836 modified the association between PD risk and smoking [31,49]. A significant interaction was observed in men but not in women [50]. Several studies, including the studies with the same data or overlapping data by the overlapping authors [23,39], also reported no interaction between *MAOB *rs1799836 and smoking in PD risk [23,35,36,39]. Other environmental factors may reduce MAOB and such phenotypic determinants may vary across populations. Given the possibility of environmental effects on MAOB activity, further work on interactions between the *MAOB *polymorphism and smoking is needed. There was no interaction between smoking and *DRD2 *rs180049 in two studies [36,39]. No studies examining the interactions between smoking and the *DRD4 *rs1800955 in PD have been published to date. Ethnicity might also play a role when studying the role of genetic factors in the association between smoking and PD.

Accumulation of multiple "at-risk" alleles markedly increased the risk of PD in a dose dependent manner, although each "at-risk" allele was associated with a small increase in risk (Table 4). Thus, individuals may have several nonsignificant "at-risk" genotypes" whose combined effect results in a high-risk. Compared with known nongenetic risk factors, smoking (1/0.40 = 2.50) and a combination of "at-risk" alleles (2.66, seven or eight "at-risk" alleles vs, less than four "at-risk" alleles) provided the same impact in PD risk prediction. Our study therefore suggests that the combined effect of multiple variant alleles may be more important than the investigation of a SNP in modulating PD risk although "at-risk" allele combinations are rare in the general population. However, although an "at-risk" allele (genotype) may confer a small individual risk, this small increase in risk translates to a large number of excess PD cases in the population. Therefore, the polymorphisms, even those not significantly associated with PD, should be considered an important public health issue.

One strength of our study is that cases were identified according to strict diagnostic criteria, and thus the possibility of misclassification of PD is negligible. Several limitations of the study also warrant mention. Our study may have included a bias due to the self-reporting of smoking habits. However, discrepancies between self-reported smoking habits and biochemical verification are minimal among the general population [51,52]. Another problem with case-control studies is recall bias. As risk factors for PD are poorly characterized, study subjects have few systematic preconceived ideas regarding their disease etiology. Any recall bias was likely to be non-differential given the many pesticides reported, the complex temporal pattern of their use, and the fact that subjects were not informed of the study hypotheses.

## Conclusions

Our results suggest that the *MAOB *rs1799836 played an important role in PD susceptibility in our Japanese population. To the best of our knowledge, this is the first report on the relationship between *DRD4 *rs1800955 with PD. Our study provides evidence of the interaction between *COMT *rs4680 polymorphisms and smoking. The previous studies have failed to confirm any significant association between PD and rs1799836/rs4680 [53], however. Replication of findings is very important before any causal inference can be drawn. In order to confirm our findings, consortia of investigators working on PD may need to be established. Future studies involving larger control and case populations and better pesticide exposure histories will undoubtedly lead to a more thorough understanding of the role of the polymorphisms involved in dopaminergic neurotransmission in PD development.

## Appendix

Other members of the Fukuoka Kinki Parkinson's Disease Study Group are as follows: Yasuhiko Baba and Tomonori Kobayashi (Department of Neurology, Faculty of Medicine, Fukuoka University); Hideyuki Sawada, Eiji Mizuta and Nagako Murase (Clinical Research Institute and Department of Neurology, Utano National Hospital); Tsuyoshi Tsutada and Takami Miki (Department of Geriatrics and Neurology, Osaka City University Graduate School of Medicine); Jun-ichi Kira (Department of Neurology, Neurological Institute, Graduate School of Medical Sciences, Kyushu University); Tameko Kihira and Tomoyoshi Kondo (Department of Neurology, Wakayama Medical University); Hidekazu Tomimoto (Department of Neurology, Kyoto University Graduate School of Medicine); Takayuki Taniwaki (Division of Respirology, Neurology, and Rheumatology, Department of Medicine, Kurume University School of Medicine); Hiroshi Sugiyama and Sonoyo Yoshida (Department of Neurology, Kyoto-Minami National Hospital); Harutoshi Fujimura and Tomoko Saito (Department of Neurology, Toneyama National Hospital); Kyoko Saida and Junko Fujitake (Department of Neurology, Kyoto City Hospital); Naoki Fujii (Department of Neurology, Neuro-Muscular Center, National Omuta Hospital); Masatoshi Naito and Jun Arimizu (Department of Orthopaedic Surgery, Faculty of Medicine, Fukuoka University); Takashi Nakagawa, Hirofumi Harada, and Takayuki Sueta (Department of Otorhinolaryngology, Faculty of Medicine, Fukuoka University); Toshihiro Kikuta and George Umemoto (Department of Oral and Maxillofacial Surgery, Faculty of Medicine, Fukuoka University); Eiichi Uchio and Hironori Migita (Department of Ophthalmology, Faculty of Medicine, Fukuoka University); Kenichi Kazuki, Yoichi Ito, and Hiroyoshi Iwaki (Department of Orthopaedic Surgery, Osaka City University Graduate School of Medicine); Kunihiko Siraki and Shinsuke Ataka (Department of Ophthalmology and Visual Sciences, Osaka City University Graduate School of Medicine); Hideo Yamane and Rie Tochino (Department of Otolaryngology and Head and Neck Surgery, Osaka City University Graduate School of Medicine); Teruichi Harada (Department of Plastic and Reconstructive Surgery, Osaka City University Graduate School of Medicine); Yasushi Iwashita, Motoyuki Shimizu, Kenji Seki, and Keiji Ando (Department of Orthopedic Surgery, Utano National Hospital).

## List of abbreviations

CI: confidence interval; CNS: central nervous system; COMT: catechol-O-methyltransferase; DRD2: dopamine receptor D2; DRD4: dopamine receptor D4; HWE: Hardy-Weinberg equilibrium; MAOB: monoamine oxidase B, OR, odds ratio; PD: Parkinson's disease; SNP: single nucleotide polymorphism.

## Competing interests

The authors declare that they have no competing interests.

## Authors' contributions

CK contributed to study design, data collection, data management, statistical analysis, data interpretation, and manuscript writing. YM contributed to study design, data collection, overall management, data interpretation, and manuscript editing. MK, TF and SS carried out performed laboratory work. KT, WF and SS contributed to study design, data collection, and data management. YT, TY, TO, HS, NK, NS, and HF contributed to outcome definition and case recruitment. YH and MN contributed to conception of the design and execution of the study. Authors listed in the Appendix contributed to case or control subject recruitment. All authors provided comments on the drafts and have read and approved the final version.

## Pre-publication history

The pre-publication history for this paper can be accessed here:

http://www.biomedcentral.com/1471-2377/11/89/prepub
